# High-Throughput Continuous-Flow Separation in a Micro Free-Flow Electrophoresis Glass Chip Based on Laser Microfabrication

**DOI:** 10.3390/s22031124

**Published:** 2022-02-01

**Authors:** Aodong Zhang, Jian Xu, Xiaolong Li, Zijie Lin, Yunpeng Song, Xin Li, Zhenhua Wang, Ya Cheng

**Affiliations:** 1 Engineering Research Center for Nanophotonics and Advanced Instrument, School of Physics and Electronic Science, East China Normal University, Shanghai 200241, China; 52210920030@stu.ecnu.edu.cn (A.Z.); zhwang@phy.ecnu.edu.cn (Z.W.); ya.cheng@siom.ac.cn (Y.C.); 2 State Key Laboratory of Precision Spectroscopy, School of Physics and Electronic Science, East China Normal University, Shanghai 200241, China; 51174700030@stu.ecnu.edu.cn (X.L.); 52170920036@stu.ecnu.edu.cn (Z.L.); 52190920011@stu.ecnu.edu.cn (Y.S.); 3 XXL—The Extreme Optoelectromechanics Laboratory, School of Physics and Electronic Science, East China Normal University, Shanghai 200241, China; 4 State Key Laboratory of High Field Laser Physics, Shanghai Institute of Optics and Fine Mechanics, Chinese Academy of Sciences, Shanghai 201800, China

**Keywords:** femtosecond laser microfabrication, glass microfluidics, laser-assisted chemical etching, micro free-flow electrophoresis, continuous-flow separation

## Abstract

Micro free-flow electrophoresis (μFFE) provides a rapid and straightforward route for the high-performance online separation and purification of targeted liquid samples in a mild manner. However, the facile fabrication of a μFFE device with high throughput and high stability remains a challenge due to the technical barriers of electrode integration and structural design for the removal of bubbles for conventional methods. To address this, the design and fabrication of a high-throughput μFFE chip are proposed using laser-assisted chemical etching of glass followed by electrode integration and subsequent low-temperature bonding. The careful design of the height ratio of the separation chamber and electrode channels combined with a high flow rate of buffer solution allows the efficient removal of electrolysis-generated bubbles along the deep electrode channels during continuous-flow separation. The introduction of microchannel arrays further enhances the stability of on-chip high-throughput separation. As a proof-of-concept, high-performance purification of fluorescein sodium solution with a separation purity of ~97.9% at a voltage of 250 V from the mixture sample solution of fluorescein sodium and rhodamine 6G solution is demonstrated.

## 1. Introduction

Micro free-flow electrophoresis (μFFE) is a versatile technique for high-efficiency continuous separation and purification of liquid analytes with an electric-field-controlled microfluidic environment [1,2,3,4,5,6]. In μFFE, the flow direction of liquid streams in a separation microchannel or micro-chamber is perpendicular to the direction of the electric field, which enables the different displacements of the pre-separated samples to realize spatial microscale separation depending on their electrical mobilities surrounded by the background solution under the electric field. Due to its remarkably continuous-flow capability of online processing, this technique has shown broad prospects in chemical, biological, and medical applications such as the high-performance purification of various fine chemicals, proteins, and biomolecules.

To date, some representative methods to prepare μFFE chips have been developed [7,8,9,10,11,12,13,14,15,16,17,18,19,20,21]. For polymeric materials such as PDMS and ABS plastics, soft-lithography [8] or three-dimensional (3D) printing [9] are popularly adopted due to their economy and ease of operation. However, for some specific applications, such as the separation of organic molecules and toxic substances, the use of glass materials such as fused silica is more desirable regarding the superior chemical inertness, wide transmission window, and mechanical strengths of glass. For fabrication of glass-based μFFE chips, usually, a combination of the lithographic method and subsequent bonding is widely used [10,11,12,13,14]. To ensure the long-term and stable operation of μFFE, the electrolytic bubbles generated around the electrodes need to be timely expelled. Otherwise, those bubbles could severely affect the stability of the separation performance. Therefore, rational design of chip configurations, including controllable isolation of micro-separation units and integrated electrodes, is of vital importance. Although direct integration of the electrodes to the sides of a separation unit without isolation can apply for the μFFE chip, the performance of the chip is inherently limited in terms of its low separation resolution and low throughput. To address the bubble issues, several protocols such as physical isolation using geometrical structures [10,14], integration of membranes [11,15,16], and gels as salt bridges [17] have been adopted in many μFFE devices. However, the manufacture of high-throughput and highly stable μFFE glass chips in a simple fashion remains a challenge in terms of tedious and complex fabrication procedures.

In this paper, a facile method for fabricating a high-throughput μFFE chip based on femtosecond (fs) laser-assisted chemical etching of glass is demonstrated. Based on the combination of merits of ultrafast laser–glass nonlinear interaction and selective chemical etching of glass, fs laser-assisted chemical etching of glass enables the versatile fabrication of 3D glass microstructures with nearly arbitrary configurations [22,23,24,25,26]. To isolate the separation chamber and electrode channels, two microchannel arrays are introduced, which can be simultaneously fabricated with other microfluidic components through fs laser microfabrication. High-throughput separation of fluorescein sodium (NaFL) and rhodamine 6G (Rh6G) surrounded by a buffer environment inside the separation chamber was demonstrated. Meanwhile, the removal of electrolysis-generated bubbles along the deep electrode channels was enhanced with a high flow rate of buffer solution. The manufactured chip can be applied to the high-throughput separation and purification of other liquid samples such as proteins and cells.

## 2. Materials and Methods

### 2.1. Chip Design

**Figure 1:**
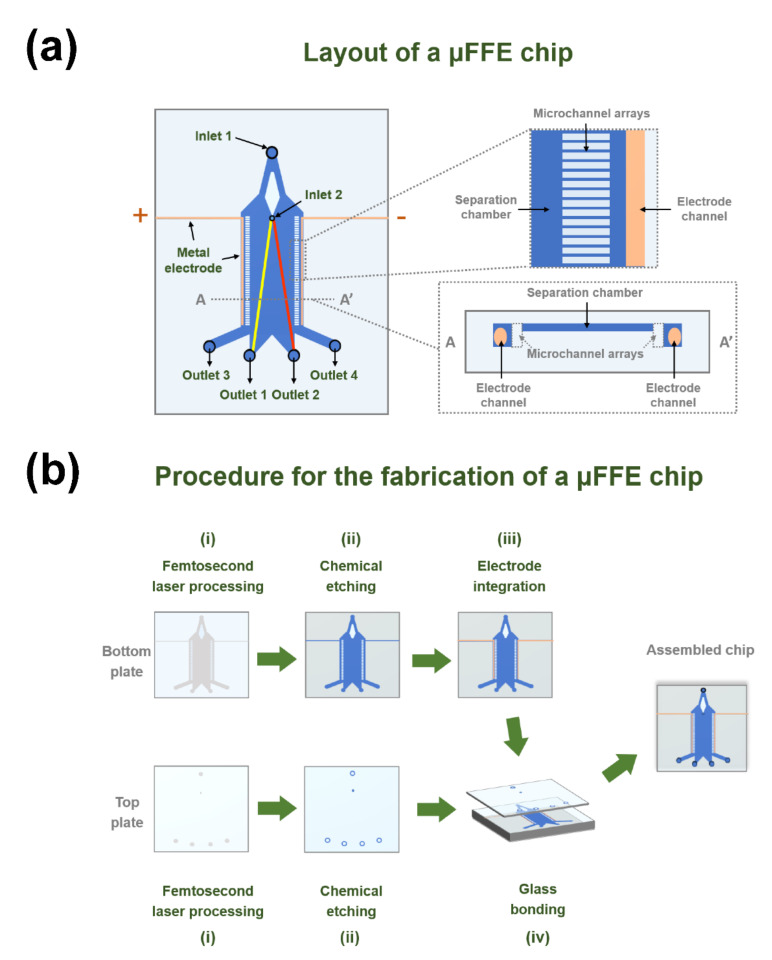
(**a**) Layout of an assembled μFFE chip. The chip consists of a separation chamber, two electrode channels, two microchannel arrays, two embedded metallic platinum electrodes, two inlets (inlet 1 for buffer solution and inlet 2 for sample solution), and four outlets (outlet 1 and outlet 2 for liquid-phase separation, outlet 3 and outlet 4 for exhaust of bubbles). (**b**) Schematic of the fabrication procedure for microscale free-flow electrophoresis (μFFE) chips based on femtosecond laser microfabrication. (i) Femtosecond laser processing of two fused silica plates, including the top and bottom plates. (ii) Chemical etching of the glass plates. (iii) Electrode integration in the bottom plate. (iv) Bonding of the plates for assembling the chip.

As illustrated in Figure 1a, the configuration of a μFFE chip in this work is inspired by the literature [9], which includes a separation chamber, two electrode channels, two microchannel arrays, two embedded metallic platinum electrodes, two inlets (inlet 1 for buffer solution and inlet 2 for sample solution), and four outlets (outlet 1 and outlet 2 for liquid-phase separation, outlet 3 and outlet 4 for exhaust of bubbles). As compared with the previous work [9,14], two microchannels arrays were introduced between the electrode channels and separation chamber to enhance the stability of the sample flow and maintain the electric-field manipulation (see the inset of Figure 1a). The height ratio of the separation chamber and the electrode channel was about 1:3.3.

### 2.2. Fabrication of a μFFE Chip

As illustrated in Figure 1b, the fabrication procedure for a μFFE chip consists of four main steps: (i) femtosecond laser processing of two fused silica plates including the top and bottom plates of the chip; (ii) chemical etching of the glass plates; (iii) electrode integration in the bottom plate; (iv) bonding of the glass plates for assembling the chip. First, fused silica glass (JGS1) with two sizes of 50 × 50 × 1 mm^3^ and 50 × 50 × 3 mm^3^ were used as the processing substrates for the fabrication of top and bottom plates of the chip, respectively. An ultrashort pulse laser amplifier system (Pharos 20 W, Light Conversion, Vilnius, Lithuania) with a central wavelength of 1030 nm, a pulse width of 270 fs, and a repetition rate of 250 kHz was employed for laser modification [26]. Both the top and bottom plates were irradiated by the focused laser beam using a 10× objective lens (M Plan Apo NIR 10X, Mitutoyo, Japan) with a numerical aperture (NA) of 0.26. The measured average power of the laser beam before the focusing optics was ~1 W. The translation speed of the plates during the processing was 30 mm/s provided by a three-dimensional (3D) programmable air-bearing stage (Aerotech X-Y-Z stage, Aerotech, Pittsburgh, PA, USA). The writing direction of the laser beam was perpendicular to the polarization direction of the beam. Since there is a tradeoff between reasonable surface quality and high fabrication efficiency for direct laser writing of centimeter-scale areas, the line-to-line spacing and the layer-to-layer spacing were set at 10 μm and 25 μm, respectively. By doing this, the laser-fabrication time for finally obtaining non-crack glass microstructures with a reasonable surface quality on the bottom plate of the chip is ~3 h.

Then, the laser-irradiated glass plates were immersed in an ultrasonic bath of 5% HF diluted solution for chemical etching until the modified regions were fully removed [22,24]. Typically, the etching of the top and bottom plates takes about 5 h and 15 h, respectively. Next, to integrate electrodes inside the chip, two metallic platinum wires with diameters of 200 μm were folded and placed into two open L-shaped electrode channels of the bottom plate, respectively, and then fixed with a silver paste (EPO-TEK^®®^ H20E; Epoxy Technology, Inc., Billerica, MA, USA). The silver paste filled the gaps between the lateral segment of the L-shaped electrode channels and the metallic wires and was subsequently solidified in an oven at 100 °C for 2 h. After that, both the top and bottom plates were coated with a thin layer of a liquid glass solution as an adhesive material (9H+; Kinilead Pro, Shenzhen, China), and further bonded with each other with a homemade fixture in an oven at 80 °C for 24 h to form an assembled μFFE chip (see Figure S1). Although the pressure-resistant performance of the device has been not measured, the leakage of the solution from the glass chip has been not yet observed in the experiments, demonstrating the validity of the low-temperature bonding process in a reproducible manner.

### 2.3. On-Chip Separation

To demonstrate on-chip separation, the buffer solution with a pH value of 7.0, which composed of 300 μM Triton X-100, 25 μM HEPES, and several drops of 1 M NaOH solution, was pumped into the inlet 1 of the μFFE chip through a piston pump (MSPF0102, Shanghai Bomo Inc., Shanghai, China). The sample solution, containing a mixture solution of 120 μM Rh6G solution and 120 μM NaFL solution, was pumped into the inlet 2 of the chip through a syringe pump (RSP01-B, Biotaor instrument, Jiaxing, China). The flow rate of the buffer solution was set at 3 mL/min, and the flow rate of the sample solution was varied from 75 to 300 μL/min. The applied electrical voltage between the electrodes was varied from 50 to 250 V. The conductivities of the sample solution and the buffer solution were measured to be ~50 and ~465 μS/cm, respectively. The solutions passing through the FFE chip were collected from outlet 1 and outlet 2 for measurement of absorption spectra, respectively. The separation purity of the chip was evaluated by the ratio of the estimated concentration of NaFL solution from outlet 1 to the sum of the estimated concentrations of NaFL solution and Rh6G solution collected from outlet 1 based on absorbance measurement.

### 2.4. Characterization

The morphology of glass samples was observed and recorded by an optical microscope (BX53, Olympus, Tokyo, Japan). The photos and videos of on-chip separation were captured by a microscope system (GP-600V; Kunshan Gaopin Inc., Kunshan, China). The conductivities of the sample solution and the buffer solution were measured by a conductivity meter (LAQUAtwin EC-33, Horiba, Japan). The optical density (OD) values of optical microscope images were analyzed using ImageJ software (NIH, Bethesda, MD, USA, https://imagej.nih.gov/ij/, accessed on 25 April 2021). The absorption spectra of the liquid samples were measured by an ultraviolet-visible (UV–Vis) spectrometer (UV-2600, Shimadzu, Kyoto, Japan).

## 3. Results

### 3.1. Femtosecond Laser-Assisted Chemical Etching of Fused Silica Glass

**Figure 2:**
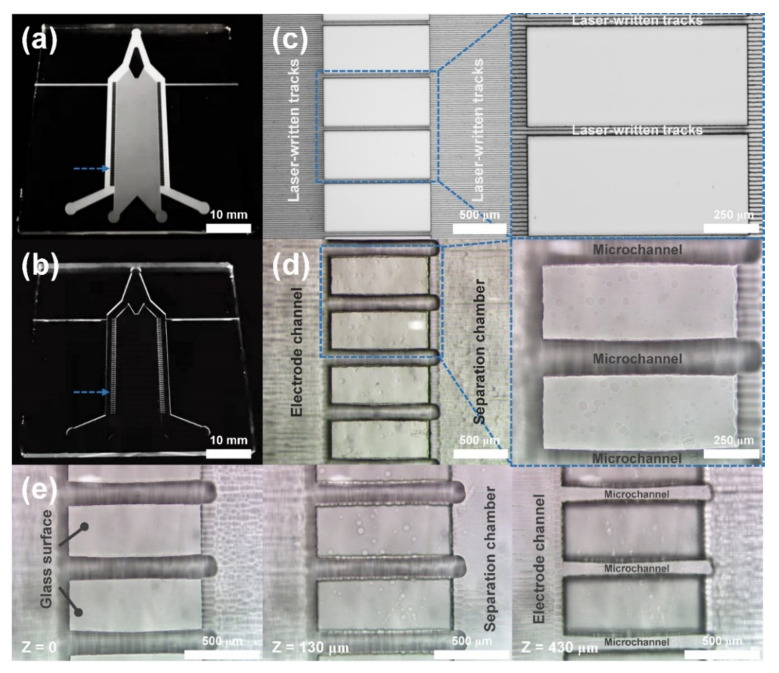
Photos of fs-laser-processed fused silica bottom plates (**a**) before and (**b**) after chemical etching. Optical microscope images of the laser-processed microstructures (**c**) before and (**d**) after chemical etching as indicated by dashed arrows in (**a**,**b**), respectively. Insets in (**c**,**d**) show close-up images of the dashed rectangular areas in (**c**,**d**), respectively. (**e**) Optical microscope images of the etched microstructures at different focal positions. Left: the glass surface; Middle: the bottom surface of the separation chamber; Right: the bottom surface of the electrode channel and microchannels.

To assemble a μFFE chip with desirable size, high-precision and high-quality fabrication of the bottom plate of the chip is of vital importance through fs laser-assisted chemical etching since the top plate of the chip was only composed of several vertical ports. Figure 2a,b show photos of fs-laser-processed fused silica samples before and after chemical etching, respectively. One can see that the laser-modified areas (see gray parts in Figure 2a) can be fully removed to form a bottom plate with open microfluidic structures (see Figure 1b and Figure 2b) after chemical etching. Figure 2c,d show optical microscope images of the laser-processed microstructures indicated by dashed arrows in Figure 2a,b, respectively. Laser written tracks after 5% HF ultrasonic etching for 15 h were removed to form open microfluidic structures, including an electrode channel, a separation chamber, and several parallel microchannels. Especially, as shown in the insets of Figure 2c,d, the microchannels with widths of ~150 μm and depths of ~430 μm were obtained through the chemical etching of laser-written tracks with widths of ~40 μm and depths of ~450 μm. The decrease of the depths, as well as the increase of the widths, may be ascribed to the spatially isotropic etching of the pristine fused silica in 15 h HF etching besides the removal of the laser-written tracks [22,24]. With the spatial control of the fs laser direct writing scheme and subsequent chemical etching, open microfluidic structures with different heights can be easily fabricated to form different parts of the bottom plate of the chip. Figure 2e show optical microscope images of the etched microstructures at different focal positions. The height of the separation chamber and the electrode channel were ~130 μm and ~430 μm (as same as the microchannel array, see the right panel of Figure 2e), respectively. In principle, the geometrical shapes and sizes of the open microfluidic structures can be controlled by tuning the processing parameters of fs laser direct writing (e.g., pulse energy, writing speed, line-by-line spacing, layer-by-layer spacing, etc.) and the subsequent chemical etching process (e.g., etching time, etc.) [22,23,24,25,26]. Herein, a height ratio of ~1:3.3 between the separation chamber and the electrode channel was optimized to enhance the exhaust of bubbles and maintain the stability of the separation processes. When the height ratios were set at 1:2 and 1:4.3, both manufactured chips could not experimentally obtain the stably electrophoretic separation. In the case of the height ratio of 1:2, the in situ generated electrolysis bubbles could not be effectively expelled due to the low flow rate of buffer solution in the electrode channels in the experiments. While in the case of the height ratio of 1:4.3, although the bubble issues could be solved, the stability of the sample flow in the separation chamber could not maintain at a high flow rate of the buffer solution in the electrode channels.

### 3.2. On-Chip Separation under Different Applied Voltages

**Figure 3:**
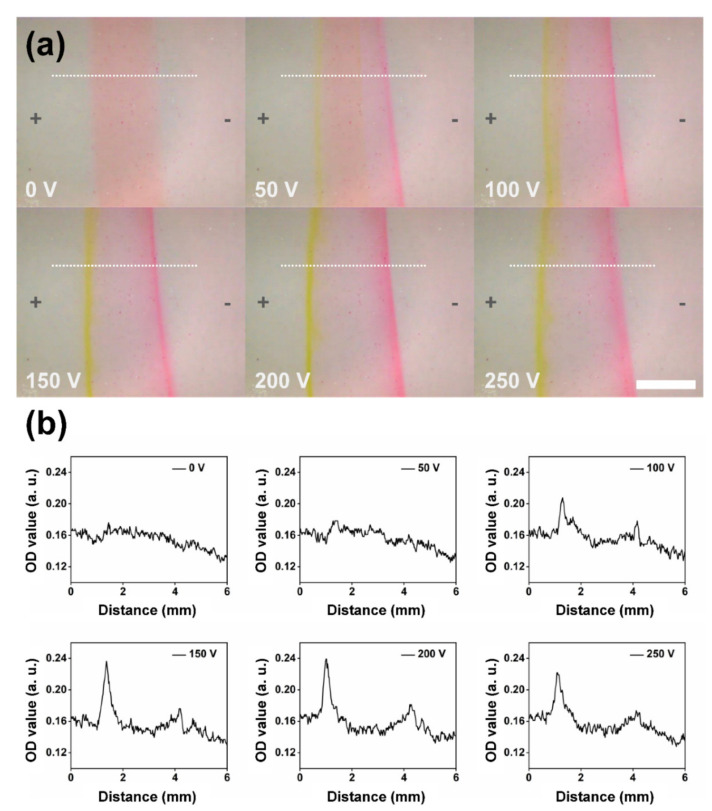
(**a**) Optical micrographs of the sample solution (the mixture solution of Rh6G and NaFL) in the separation chamber under different applied voltages ranging from 0 V to 250 V. The direction of the electric field was from left (+) to right (−). The flow rates of sample solution and buffer solutions were set at 200 μL/min and 3 mL/min, respectively. Scale bar: 2.5 mm. (**b**) Optical density (OD) value versus the horizontal distance along the separation chamber estimated from the dashed line in (**a**) with different applied voltages.

According to the μFFE separation theory [5,27], the lateral migration distance of the analyte (*d*) is determined by the total mobility of the analyte (*μ_total_*), the electric field in the separation chamber (*E*), and the time of the analyte in the electric field (*t*): *d* = *μ_total_* × *E* × *t*. When the proper electric field was applied perpendicularly across the separation chamber for a certain time, the positively charged Rh6G molecules and the negatively charged NaFL molecules in the buffer solutions exhibited different deflections towards the cathode and anode due to their different mobility, respectively. In the fabricated μFFE device, experimentally, both the migration distances of the NaFL and Rh6G molecules allow the controllable on-chip separation of NaFL and Rh6G from outlet 1 and outlet 2, respectively. To investigate the performance of on-chip separation under different applied voltages, the buffer and sample solutions including Rh6G and NaFL were introduced into the inlet 1 and inlet 2 with flow rates of 3 mL/min and 200 μL/min, respectively. Figure 3a show optical micrographs of the sample streams flowing in the separation chamber under different applied voltages ranging from 0 V to 250 V. When the electric field was not applied, the sample solution exhibited a wide homogeneous stripe in pink surrounded by the transparent buffer solution. When a voltage of 50 V was applied, small yellow and red stripes appeared at both edges of the whole stripe, which indicated the bidirectional migration of the positive-charge Rh6G molecule and the negative-charge NaFL molecule under the electrical field. With the increase of the applied voltages from 50 V to 200 V, the colors of the stripes become clearer, indicating the enhancement of the bidirectional migration for on-chip separation. When the applied voltages increased to 250 V, although the colors and widths of the strips slightly changed, probably due to the disturbance of continuously increased bubbles in the electrode channel, the bidirectional migration remained. Further increase of the applied voltage will lead the separation strip to become unstable.

To analyze the performance of on-chip separation, the OD values of the dashed lines with lengths of 6 mm indicated in Figure 3a were investigated. As shown in Figure 3b, the distribution of OD values along the dashed lines in Figure 3a varied at different applied voltages. As compared with the case of no electrical field, two peaks (left peak: NaFL, right peak: Rh6G) appeared when the voltages ranging from 50 V and 250 V were applied, verifying the functionality of on-chip separation. With the increase of applied voltages from 50 V to 200 V, the intensity of the peaks increased, and the distances between the peaks slightly increased due to the enhancement of the electric field. Again, when a voltage of 250 V was applied, the intensity of the peaks slightly decreased, which was consistent with the observation result in Figure 3a.

**Figure 4:**
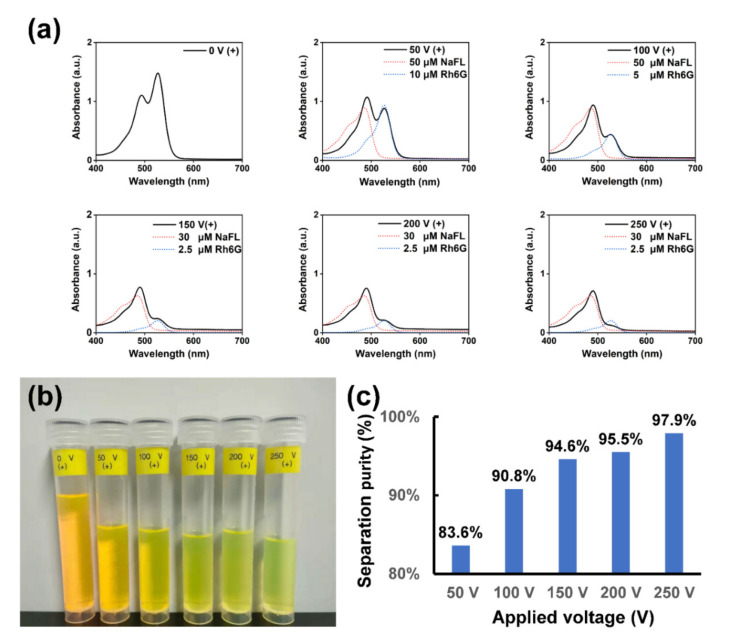
(**a**) Absorption spectra (solid curves) of the products collected from outlet 1 (near the positive electrode) with different applied voltages ranging from 0 V to 250 V. Blue and red dashed curves are absorption spectra of the Rh6G solution and the NaFL solution with different concentrations for reference, respectively. (**b**) Photo of the products collected from outlet1 (near the positive electrode) with different applied voltages. (**c**) Estimated separation purity versus applied voltages.

Herein, the separation purity was defined as the ratio of the concentration of NaFL solution (*C_NaFL_*) to the concentration of the whole solution (*C_NaFL_* + *C_Rh6G_*) in outlet 1 [28,29]. First, absorption spectra of freshly prepared standard solutions of Rh6G and NaFL with different concentrations were experimentally measured (see Figure S2a,b). Then, the calibration curves of the absorbance of Rh6G and NaFL solutions at different concentrations were plotted according to Lambert–Beer’s law based on maximum absorption peaks of Rh6G at 526 nm and NaFL at 488 nm (see Figure S2c,d). Next, the absorption spectra of the solutions collected from outlet 1 under different applied voltages were measured as shown in Figure 4a. By reading out the values of absorbance of the solutions at maximum absorption wavelengths, the concentrations of Rh6G and NaFL of the collected solutions at different applied voltages can be estimated through the calibration curves, respectively (see Table S1). Finally, the estimated separation purity was obtained. For reference, absorption spectra of standard solutions of Rh6G and NaFL with specific concentrations were presented (see Figure 4a). As shown in Figure 4b, the solutions collected from outlet 1 (near the side of the positive electrode channel), with the increase of applied voltages from 0 V to 250 V, the color turned from yellow to green, which reveals a strong voltage dependence of the separation. Figure 4c demonstrate a histogram of estimated separation purities at different applied voltages. When a voltage of 50 V was applied, the separation purity was ~83.6%. Further increase of the applied voltage leads to increased separation purity. Especially when applied voltage was set at 200 V, a separation purity of 95.5% could be obtained due to the enhancement of electrophoretic forces. It should be mentioned that, although the separation performance of 250 V seemed to be a little bit weaker than that of 200 V as shown in Figure 3, the estimated separation purity (97.9%) was higher than that of 200 V.

### 3.3. On-Chip Separation under Different Flow Rates

**Figure 5:**
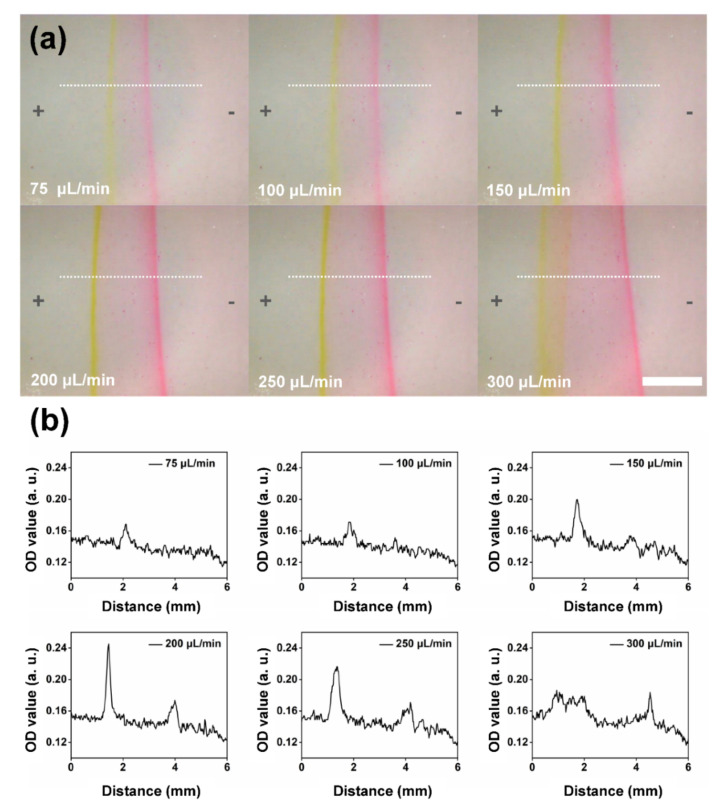
(**a**) Optical micrographs of the sample solution (the mixture solution of Rh6G and NaFL) in the separation chamber at an applied voltage of 150 V with different flow rates of the sample solutions ranging from 75 μL/min to 300 μL/min. The flow rate of the buffer solution was set at 3 mL/min. The direction of the electric field was from left (+) to right (−). Scale bar: 2.5 mm. (**b**) Optical density (O.D.) value versus the horizontal distance along the separation chamber estimated from the dashed line in (**a**) with different flow rates of the sample solutions.

Besides the effects of applied voltages, the flow rates also affected the separation performance of the chip. When the electric field was not applied, with the increase of the flow rate of the sample stream, the width of the sample stream increased (see Figure S3). As described in Figure 4c, a separation purity of 94.6% can be obtained at a voltage of 150 V when the flow rate of the sample stream was 200 μL/min. To evaluate the separation performance under different flow rates, the voltage of 150 V was also applied. Figure 5a show optical micrographs of the sample streams flowing in the separation chamber at different flow rates ranging from 75 μL/min to 300 μL/min. In all cases, the flow rates of the buffer solution and the applied voltages were 3 mL/min and 150 V, respectively. With the increase of the flow rate of the sample stream, the width of the sample stream increased as well as the throughput of the separation (see the color of two edges of the sample stream in Figure 5a). However, when the flow rate of the sample stream was 300 μL/min, two edges of the sample stream became unstable compared with the case of 250 μL/min. Therefore, there is a tradeoff between high purity and high throughput. Again, OD values of the dashed lines with lengths of 6 mm indicated in Figure 5a were analyzed. As shown in Figure 5b, the distribution of OD values along the dashed lines in Figure 5a varied at different flow rates of the sample streams. When a flow rate of 75 μL/min was adopted, the peaks of both NaFL and Rh6G were not obvious. When the flow rate increased to 200 μL/min, both the peaks reached the maximum value, indicating the enhancement of the throughput. With further increase of the flow rate, those peaks tended to decrease due to the limitation of the intensities of applied electric fields at a constant voltage of 150 V.

**Figure 6:**
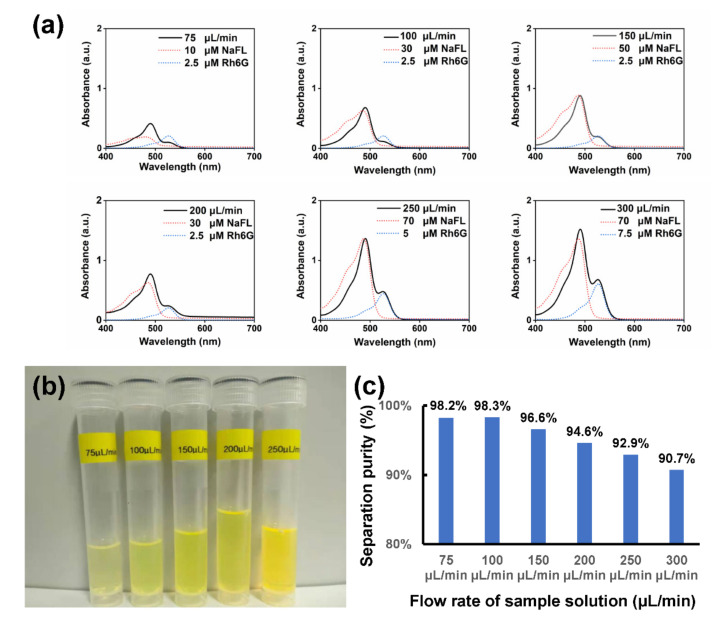
(**a**) Absorption spectra (solid curves) of the products collected from outlet 1 (near the positive electrode) with different flow rates of the sample solutions ranging from 75 μL/min to 300 μL/min. Blue and red dashed curves are absorption spectra of the Rh6G solution and the NaFL solution with different concentrations for reference, respectively. (**b**) Photo of the products collected from outlet 1 (near the positive electrode) with different flow rates of the sample solutions. (**c**) Estimated separation purity versus flow rate of the sample solution.

To further analyze the separation performance, the absorption spectra of the solutions collected from outlet 1 under different flow rates of the sample streams were measured, as shown in Figure 6a. By reading out the values of absorbance of the solutions, the concentrations of Rh6G and NaFL of the collected solutions can also be estimated through the calibration curves, respectively (see Table S2). In addition, absorption spectra of standard solutions of Rh6G and NaFL with specific concentrations were presented for reference (see Figure 6a). As shown in Figure 6b, the solutions collected from outlet 1, with the increase of flow rate of the sample stream, the color turned from green to yellow, which reveals the degradation of the separation purity as well as a tradeoff between high purity and high throughput separation. Figure 6c present a histogram of estimated separation purities at different flow rates. When flow rates of 75 μL/min and 100 μL/min were chosen, the separation purity was ~98.2% and 98.3%, respectively. Further increase of the flow rate would enhance the throughput of separation while leading to a slight decrease of the separation purity. When the flow rate of the sample stream was set at 300 μL/min, a separation purity of 90.7% was obtained at the applied voltage of 150 V. To maintain the balance between high-purity and high-throughput separation, the further increase of the electric field shown in Figure 3 could be considered a possible method (see Figure S4).

## 4. Discussion

Careful design and manufacture of the sizes and configurations of microfluidic components in a μFFE chip are of vital importance for ensuring the highly stable on-chip separation in a controllable manner. As we mentioned above, for the efficient removal of electrolytic bubbles during separation, the performance of a 1:3.3 height ratio of the separation chamber and electrode channels is superior to those of 1:2 and 1:4.3. Meanwhile, the introduction of two microchannel arrays in a μFFE chip provides more controllable separation with long-term stability due to spatial modulation of flow velocities inside the separation chamber and electrode channels. As compared with a μFFE chip without microchannel arrays, the introduction of microchannel arrays enables a shrinkage of the stripe of the sample stream in the separation chamber and further increases the stability of separation. The width of the sample stream in the chip with microchannel arrays is ~2.9 mm, which is nearly half of that of the sample stream in the chip without microchannel arrays (~5.7 mm) at the same conditions (see Figure S5). Moreover, when the applied voltages increased from 150 V to 250 V, the shift of the sample stream in the chip without microchannel arrays towards the positive electrode occurred (see Figure S6) due to the bubbles generated around the negative electrode. In contrast, the position of the sample stream in the chip with microchannel arrays almost remained stable in the same conditions (see Figure 3a).

Besides controllable separation with a high-purity and long-term stability, the throughput of separation products is also important for evaluating the performance of the μFFE chip. Herein, some representative methods for manufacturing μFFE chips are summarized in Table 1. With a 130 μm height separation chamber and a 430 μm height electrode channel, the flow rate of a sample stream in a fabricated μFFE chip using the proposed technique could reach 300 μL/min. Compared with previous works [9,14,27] for controlling bubbles based on the combination of the separation channel and the electrode channel, the introduction of microchannel arrays using the proposed methods allows stable on-chip separation in a high-throughput fashion. Further optimization of chip structures such as inner height ratios of separators and the electrodes as well as the spatial sizes of microchannel arrays, multilayer parallel, and cascaded integration of separators for the continuous increase of the throughput of high-purity microscale separation will be performed in the near future.

## 5. Conclusions

We demonstrate a μFFE chip with a high throughput manufactured by fs laser-assisted chemical etching of fused silica followed by electrode integration and subsequent low-temperature bonding with liquid glass as an adhesive layer. A 1:3.3 height ratio of the separation chamber and electrode channels combined with a 3 mL/min flow rate of buffer stream allows effective exhaust of bubbles in the electrolytic process of the solutions. The introduction of microchannel arrays between the separation chamber and electrode channels further improves the stability of the μFFE chip for controlling the removal of bubbles and maintaining the electric-field intensity. The demonstration of proof-of-concept separation of NaFL and Rh6G in fabricated chips showed great potential for the high-purity separation of chemicals and biological molecules. As compared with the conventional microfabrication methods of μFFE devices, the proposed approach has several distinct advantages. Firstly, it enables the maskless and flexible fabrication of various glass channel structures with different aspect ratios and nearly arbitrary configurations. Secondly, the fabricated glass μFFE devices allow high purity separation with high throughput as described above. Finally, low-temperature bonding using liquid glass can be performed in a controllable and reproducible manner for ensuring the long-term stability of the device. Further combination of fast micro-reaction units and optoelectronic detecting/sensing components using femtosecond laser integration technology will pave the way for the rapid manufacturing of customized glass-based factory-on-a-chip microfluidic systems.

## Figures and Tables

**Table 1:** Typical methods for the manufacture of μFFE chips.

Manufacturing Method	Flow Rate of the Sample Stream	Electric-Field Intensity	Height of Separation Chamber	Height of Electrode Channel
Soft lithography of PDMS [8]	2 μL/min	~137 V/cm	13 μm	13 μm
3D printing of ABS plastics [9]	1 μL/min	~100 V/cm	80 μm	345 μm
Lithographic processing of borosilicate glass followed by glass/glass bonding [11]	0.18 μL/min	~250 V/cm	15 μm	15 μm
Lithographic processing of Pyrex glass followed by glass/glass bonding [12]	3 μL/min	~61 V/cm	116 μm	116 μm
Lithographic processing of Borofloat33 glass followed by glass/glass bonding [13]	0.1 μL/min	~150 V/cm	10 μm	10 μm
Lithographic processing of Borofloat glass followed by glass/glass bonding [14]	0.21 μL/min	~264 V/cm	20 μm	78 μm
PDMS/glass bonding [30]	0.33 μL/min	~140 V/cm	50 μm	50 μm
This work	300 μL/min	~192 V/cm	130 μm	430 μm

## Data Availability

The data presented in this study are available on request from the corresponding author.

## Supplementary Materials

The following supporting information can be downloaded at: https://www.mdpi.com/article/10.3390/s22031124/s1, Figure S1: Photograph of a glass-based μFFE chip; Figure S2: Absorption spectra (a,b) and absorbance curves (c,d) of Rh6G and NaFL solutions at different concentrations; Figure S3: Optical micrographs of the sample solution in the separation chamber at different flow rates of the sample solutions; Figure S4: Optical micrographs of the sample solution in the separation chamber at an applied voltage of 250 V with different flow rates of the sample solutions; Figure S5: Optical micrographs of the sample solution in the separation chamber (a) without and (b) with microchannel arrays; Figure S6: Optical micrographs of the sample solution in the separation chamber without microchannel arrays at different applied voltages; Table S1: Estimated concentrations of separated Rh6G and NaFL solutions at different applied voltages; Table S2: Estimated concentrations of separated Rh6G and NaFL solutions at different flow rates of sample solutions.
