# Supplementary material for: High-Throughput Continuous-Flow Separation in a Micro Free-Flow Electrophoresis Glass Chip Based on Laser Microfabrication
Source: Sensors (Basel). 2022 Feb 1;22(3):1124. doi: 10.3390/s22031124 (PMC8838507; doi:10.3390/s22031124)
Supplement: Supplementary file 1 [file sensors-22-01124-s001.zip › sensors-1539345-supplementary.pdf]

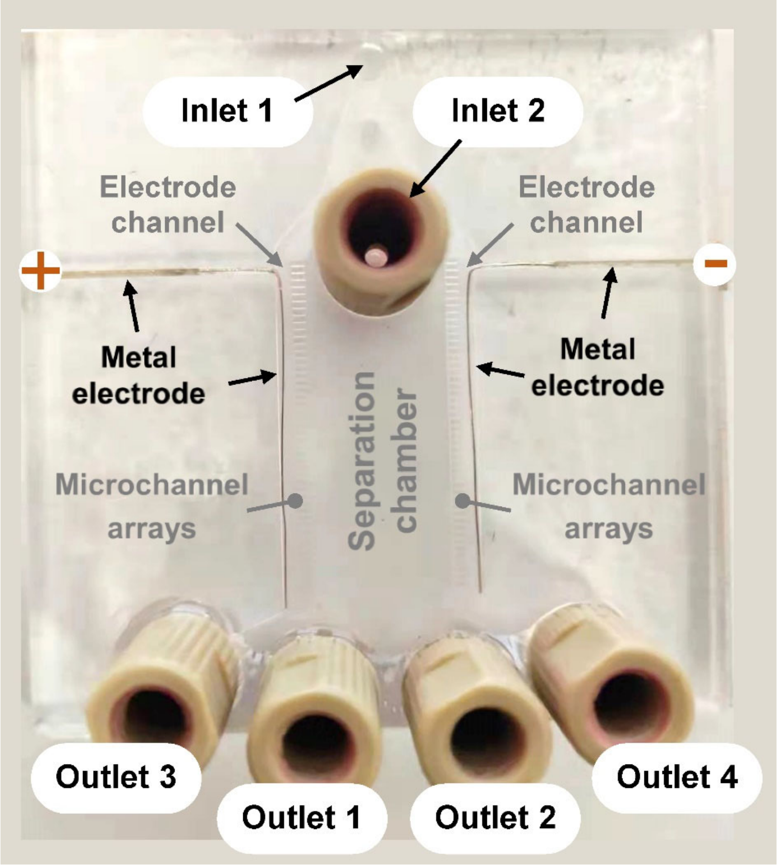

Figure S1. Photograph of a glass-based μFFE chip.

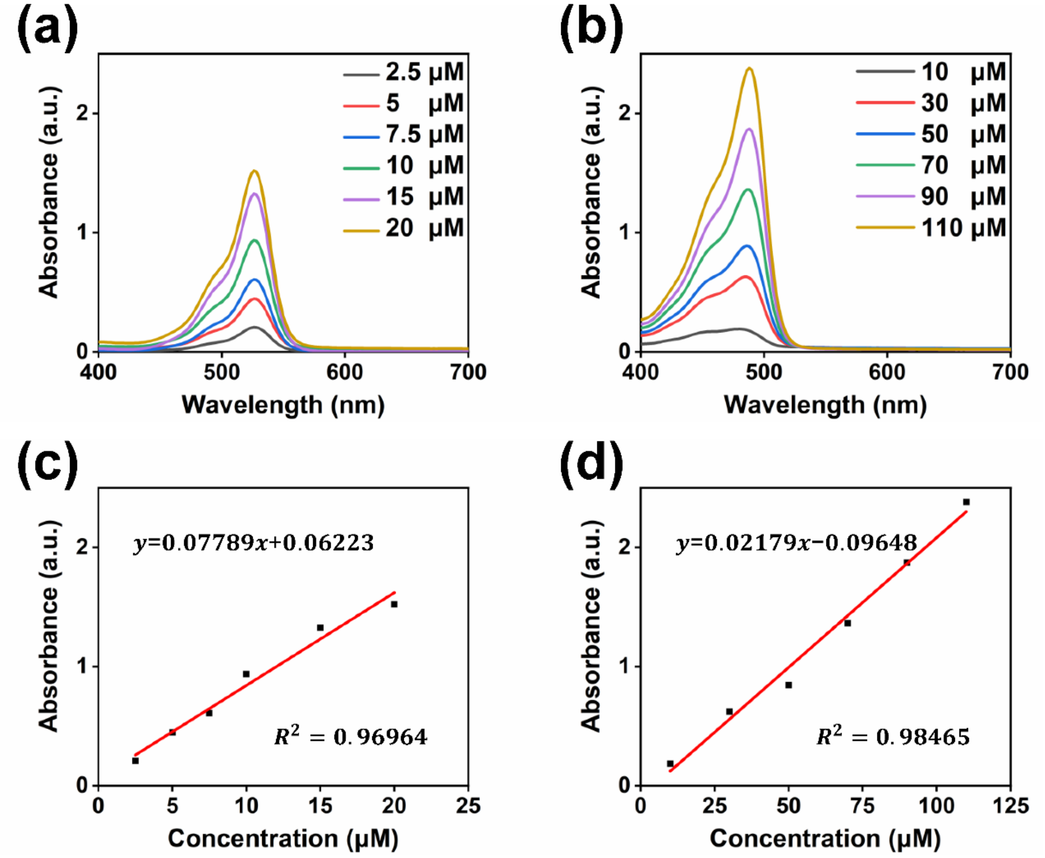

Figure S2. Absorption spectra of (**a**) Rh6G and (**b**) NaFL solutions at different concentrations. Absorbance curves of (**c**) Rh6G and (**d**) NaFL solutions at different concentrations.

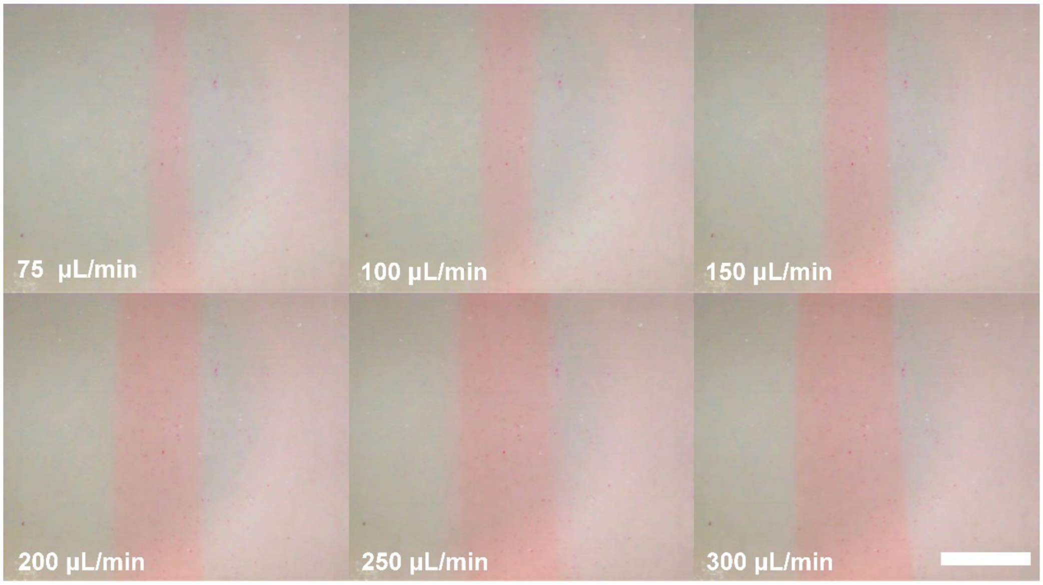

Figure S3. Optical micrographs of the sample solution in the separation chamber at different flow rates of the sample solutions ranging from 75 μL/min to 300 μL/min. The flow rate of the buffer solution was set at 3 mL/min. Scale bar: 2.5 mm.

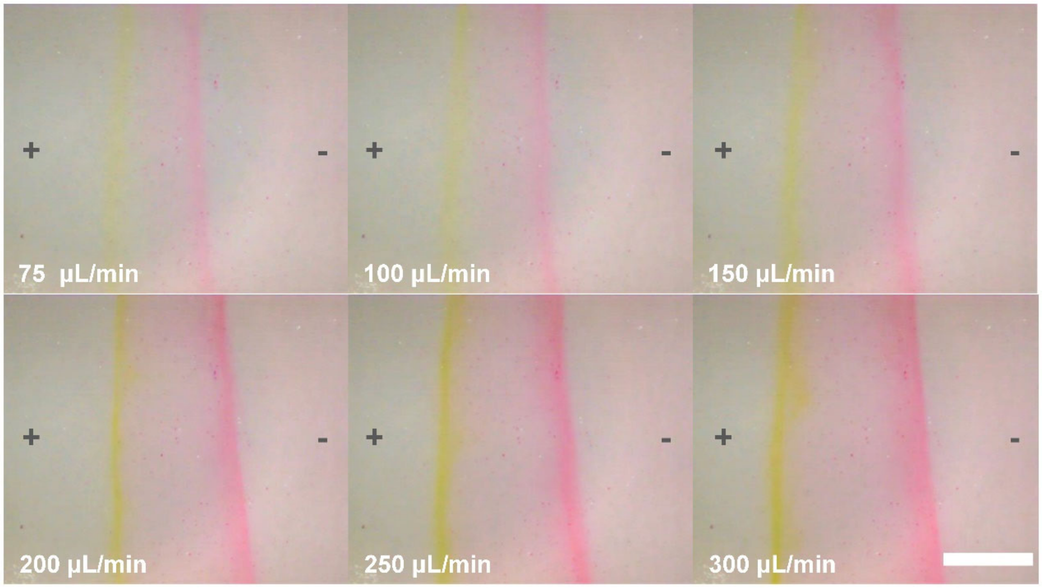

Figure S4. Optical micrographs of the sample solution in the separation chamber at an applied voltage of 250 V with different flow rates of the sample solutions ranging from 75 μL/min to 300 μL/min. The flow rate of the buffer solution was set at 3 mL/min. The direction of the electric field was from left (+) to right (-). Scale bar: 2.5 mm.

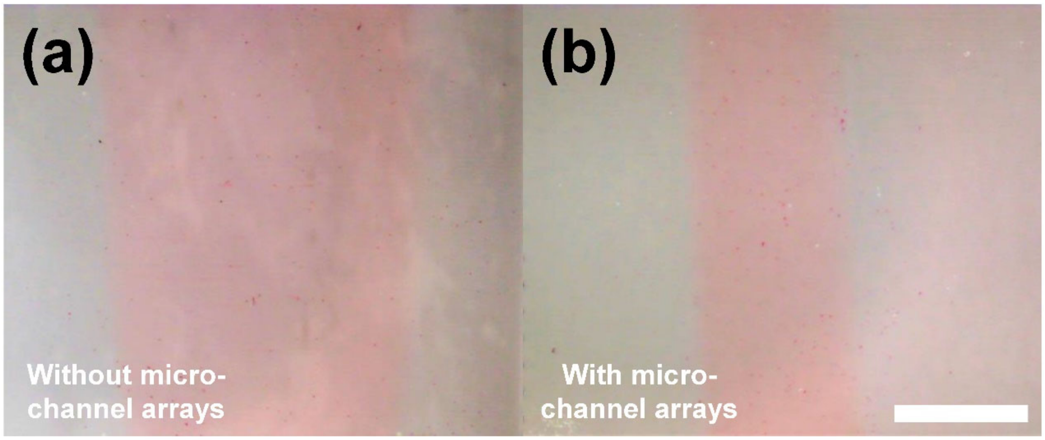

Figure S5. Optical micrographs of the sample solution in the separation chamber (a) without and (b) with microchannel arrays. The average widths of the sample stream in (a) and (b) were ~5.7 mm and ~2.9 mm, respectively. Both widths of the separation chamber in (a) and (b) are 10 mm. The flow rates of the sample solution and the buffer solution were set at 200 μL/min and 3 mL/min, respectively. Scale bar: 2.5 mm.

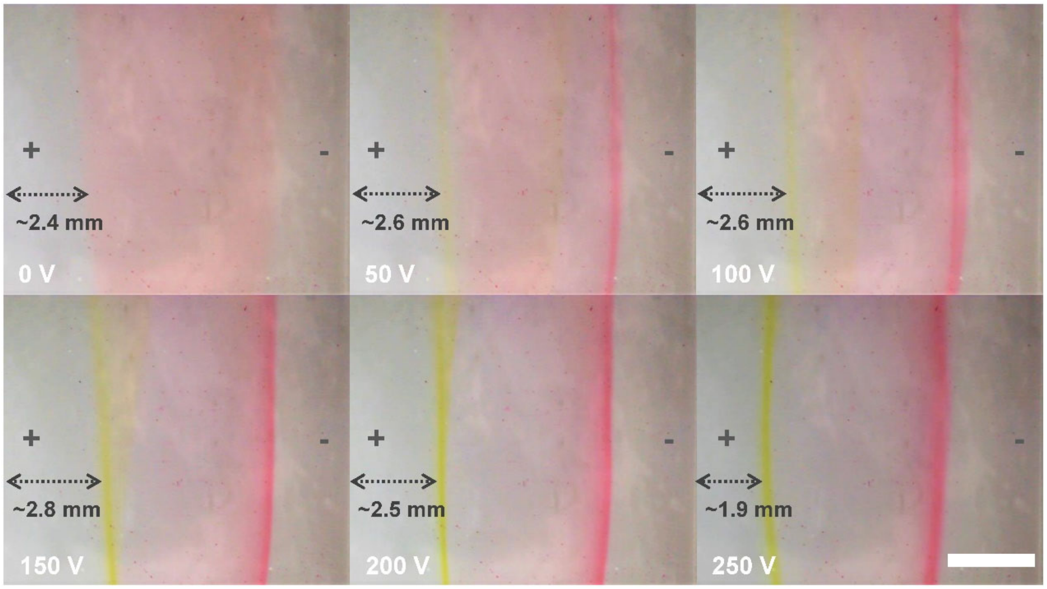

Figure S6. Optical micrographs of the sample solution in the separation chamber without microchannel arrays at different applied voltages ranging from 0 V to 250 V. The flow rates of the sample solution and the buffer solution were set at 200 μL/min and 3 mL/min, respectively. The direction of the electric field was from left (+) to right (-). With the increase of applied voltages from 150 V to 250 V, the sample stream shifted toward the positive electrode. Scale bar: 2.5 mm.

Table S1. Estimated concentrations of separated Rh6G and NaFL solutions collected from outlet 1 at different applied voltages.

Applied Voltage (V)	Concentration of Rh6G Solution (μM)	Concentration of NaFL Solution (μM)
50	10.524	53.532
100	4.812	47.56
150	2.269	39.994
200	1.871	39.214
250	0.780	37.149

Table S2 Estimated concentrations of separated Rh6G and NaFL solutions collected from outlet 1 at different flow rates of sample solutions.

Flow rate of sample solution (μL/min)	Concentration of Rh6G solution (μM)	Concentration of NaFL solution (μM)
75	0.434	23.473
100	0.600	35.772
150	1.576	44.997
200	2.269	39.994
250	5.107	67.209
300	7.598	74.322
